# Associations of Breast Cancer Risk Factors with Premenopausal Sex Hormones in Women with Very Low Breast Cancer Risk

**DOI:** 10.3390/ijerph13111066

**Published:** 2016-10-31

**Authors:** Lauren C. Houghton, Davaasambuu Ganmaa, Philip S. Rosenberg, Dambadarjaa Davaalkham, Frank Z. Stanczyk, Robert N. Hoover, Rebecca Troisi

**Affiliations:** 1 Department of Epidemiology, Mailman School of Public Health, Columbia University, New York, NY 10032, USA; lh2746@columbia.edu; 2 Channing Division of Network Medicine, Department of Medicine, Brigham and Women’s Hospital and Harvard Medical School, Department of Nutrition, Harvard T.H. Chan School of Public Health, Boston, MA 02115, USA; gdavaasa@hsph.harvard.edu; 3 Division of Cancer Epidemiology and Genetics, National Cancer Institute, National Institutes of Health, Bethesda, MD 20892, USA; rosenbep@exchange.nih.gov (P.S.R.); hooverr@mail.nih.gov (R.N.H.); 4 Health Sciences University of Mongolia, Ulaanbaatar 210648, Mongolia; davaalham@yahoo.com; 5 Division of Reproductive Endocrinology and Infertility, Keck School of Medicine of the University of Southern California, Los Angeles, CA 90033, USA; fstanczyk@att.net

**Keywords:** breast cancer, sex steroids, estrogen, progesterone, testosterone, urban migration, Mongolia

## Abstract

Breast cancer incidence rates are low but rising in urban Mongolia. We collected reproductive and lifestyle factor information and measured anthropometrics and serum sex steroid concentrations among 314 premenopausal women living in Ulaanbaatar, Mongolia. Mean differences in hormone concentrations by these factors were calculated using age-adjusted quadratic regression splines. Estrone and estradiol in college-educated women were, respectively, 18.2% (*p* = 0.03) and 23.6% (*p* = 0.03) lower than in high-school-educated women. Progesterone concentrations appeared 55.8% lower (*p* = 0.10) in women residing in modern housing compared with women living in traditional housing (gers), although this finding was not statistically significant. Testosterone concentrations were positively associated with adiposity and central fat distribution; 17.1% difference (*p* = 0.001) for highest vs. lowest quarter for body mass index and 15.1% difference (*p* = 0.005) for waist-to-height ratio. Estrogens were higher in the follicular phase of women who breastfed each child for shorter durations. A distinct hormonal profile was associated with an urban lifestyle in premenopausal, Mongol women. In particular, heavier, more-educated women living in urban dwellings had higher testosterone and lower estrogen and progesterone levels. Higher breast cancer incidence in urban compared with rural women suggest that the hormonal profile associated with a more traditional lifestyle may be protective among Mongol women.

## 1. Introduction

The near six-fold difference in breast cancer incidence worldwide, with some of the highest rates in western Europe and some of the lowest in eastern Asia [1], continues to be a rich area for investigation of underlying biological mechanisms in breast carcinogenesis. For example, Mongolia has one of the lowest breast cancer incidence rates in the world, approximately one-tenth of the UK (estimated age-standardized incidence rates (ASR) for breast cancer in 2012 were 95 per 100,000 person-years in the UK and 9.4 per 100,000 in Mongolia) [1], and rates appear to be higher in urban than in rural women [2]. While much of the difference in rates between less and more developed countries is in postmenopausal breast cancer, premenopausal incidence rates in China, for example, remain almost a third of those in the UK [3]. When Asian women migrate to the USA, their risk for breast cancer increases substantially [4], raising speculation that factors associated with living in an economically developed country may be important, including increased height, fewer children and older age at first pregnancy, changing infant feeding patterns, increasing rates of obesity, less physical activity, and dietary changes.

In general, breast cancer risk factors are hypothesized to operate through hormonal mechanisms. Testosterone concentrations appear to be higher in premenopausal breast cancer cases than controls in both Western and Asian populations [5,6,7,8], whereas there is inconsistent evidence regarding whether estrogen concentrations are positively associated with premenopausal breast cancer in either Western [6,9] or Asian populations [7,8,10]. Lower estrogen concentrations in Chinese and Japanese women compared with USA and UK women [10,11,12] have led to the hypothesis that they explain the substantial difference between Western and Asian breast cancer incidence rates. However, the observed differences in estrogen concentrations are not large enough to account for the premenopausal breast cancer incidence rate difference between these countries [3,13]. Moreover, most studies have been conducted in China and Japan, with little representation of other Asian populations.

Eastern Asia is a large, geographically and culturally diverse area with important differences in lifestyle and diet. For example, while Mongolia’s breast cancer incidence rate is at least as low as, and possibly lower than, rates in other parts of Asia [2], their dietary reliance on meat and dairy is unusual among many Asian countries [14]. Furthermore, breast cancer incidence rates within Mongolia are rising and appear to be higher in urban than rural areas [2]. We recently demonstrated evidence of elevated estrogens and reduced testosterone concentrations in premenopausal Mongol women compared with premenopausal British women [15]. Progesterone concentrations also were higher among Mongol women when compared with British women, especially in women who were born in the Mongolian countryside. This raises the possibility that changes in lifestyle associated with urban migration in Mongolia, namely, from a lifestyle that is traditional to an urban one, are associated with a decline in progesterone levels and possibly, an increase in premenopausal breast cancer risk.

In this exploratory analysis, we describe the associations of circulating sex hormones with lifestyle, reproductive, and anthropometric characteristics in a sample of premenopausal Mongol women—a unique, low breast cancer risk population.

## 2. Materials and Methods

### 2.1. Study Population

The current analysis uses data from Mongol women who participated in a larger study designed to assess the differences in hormones between premenopausal women living in Mongolia and in the UK [15]. Study participants were the mothers of school children attending two primary schools in Ulaanbaatar, Mongolia’s capital city and the most population-dense area in a country whose population is mostly nomadic. One school was located in the city center, which tended to include women born in Ulaanbaatar, and the other was located in the city’s outskirts where recent migrants from the countryside tend to settle. Data were collected in half-day sessions in April and May, 2009. Eligible participants were mothers of children attending grades 1 through 4, were 45 years of age or younger, and were not pregnant or breastfeeding and still menstruating. Of the 420 invited women, 100% agreed to participate; 221 from the school in the city center, and 199 from the school on the city’s outskirts. Women currently using oral contraceptives were excluded from the hormone analyses, leaving 137 women born in Ulaanbaatar (57% recruited from the city center) and 177 women born outside of Ulaanbaatar (born in all but 2 of the country’s 22 provinces and 53% recruited from the city center). Ethical review boards at Mongolia’s Ministry of Health, the National University of Mongolia, the United States National Cancer Institute (National Institutes of Health), and the Harvard School of Public Health approved the study (IRB#09CN078). All participants provided written, informed consent.

### 2.2. Data Collection

Trained study staff interviewed participants about demographics and migration, and medical and reproductive history. Specific information was obtained regarding birth place, lifetime migration experience, and housing (living in traditional housing (i.e., ger) vs. house or apartment), as well as smoking status (ever vs. never, because only 12 of the ever smokers were former smokers), alcohol intake and physical activity. Leisure and work-related physical activity was assessed with the Global Physical Activity Questionnaire, developed by the World Health Organization (WHO), which collects information about physical activity at work, during travel, and during recreation. It has been shown to be valid and reliable in different cultural settings [16]. The intensity of activity was coded as low, moderate, or vigorous [16]. Participants were asked about age at menarche, number of births, and breastfeeding duration for each birth. Trained personnel made standardized measurements of weight, height and waist circumference at the time of interview. Weight at 18 years of age was also asked during the interview. Body mass index (BMI) was calculated as weight divided by squared height, and the ratio of waist circumference to height was calculated.

Whole blood was collected at the time of interview and immediately delivered to the laboratory in Ulaanbaatar where blood was allowed to clot at room temperature. Samples were centrifuged and sera were stored at −70 °C until shipped to the biorepository in the USA Blood collection was not timed with regard to menstrual cycle; participants reported the date of their last menstrual period.

### 2.3. Hormone Assays

Hormones were measured in serum at the Reproductive Endocrine Research Laboratory at the University of Southern California, Keck School of Medicine under direct supervision (FZS). Concentrations of estrone, estradiol, testosterone, and androstenedione were measured by radioimmunoassay (RIA) following extraction with organic solvent and purification by Celite column partition chromatography as described previously [17,18,19]. Since progesterone coelutes in the same fraction as androstenedione on the Celite column, it was processed separately in the same manner as the other steroids. The laboratory technicians were blind to quality control samples, which constituted 10% of the batch samples. The interassay coefficients of variations were 6.2% for androstenedione, 8.2% for testosterone, 6.3% for estradiol, 4.9% for estrone, and 15.0% for progesterone. The assay sensitivities were 0.03 ng/mL, 0.015 ng/mL, 2 pg/mL, 4 pg/mL, and 0.01 ng/mL, respectively. Values for estrone and estradiol were several standard deviations (SD) beyond the next highest values for two of the women and were excluded from analyses. 

### 2.4. Statistical Analysis

Generalized additive models [20] estimated mean differences in hormone concentrations between lifestyle, reproductive, and anthropometric categories. In this exploratory study, we assessed associations between five hormones and 17 risk factors. No adjustments were made for multiple testing. Categories for most factors (including age, height, weight, waist circumference, waist-to-height ratio, BMI, weight at age 18, BMI at age 18, breastfeeding, and age at menarche) were based on the highest quartile, the combined middle two quartiles, and the lowest quartile to test whether different factors were associated with very high hormone concentrations (top 25%) or very low hormone concentrations (bottom 25%), while other categorical variables were constructed as follows: parity (1 vs. >1), education (high school vs. college), house type (ger vs. house or apartment), previously lived as a nomad (yes vs. no), physical activity (low, moderate, vigorous), current alcohol consumption (yes vs. no), and ever smoker (yes vs. no). 

Because blood collection was not timed with regard to menstrual cycle day, the hormone values represent random (but known) days since the last menstrual period (LMP). For analysis, the menstrual cycle was truncated at 28 days because of small numbers of women with longer cycles (*n* = 3). Out of the women who reported a LMP, 53% provided samples during the follicular phase. Systematic variation according to the number of days since LMP was modeled using quadratic regression splines with evenly spaced knots; the number of segments was selected using the Akaike information criterion (AIC) for all the women combined without any adjustment for covariate effects [20]. Two age-adjusted spline models were fitted for each hormone: a main effects model with a common set of coefficients for the splined effects of days since LMP, and an interaction model with separate spline coefficients for each category of explanatory variables. The interaction model was used to test whether the pattern of a hormone level over days since LMP was the same or different within each level of a covariate, (i.e., whether the splined effect of LMP was parallel within strata or interacts (varies) by strata). When there is little or no evidence for any interaction, the covariate effects from the main effects model provides a valid summary of how the mean hormone level varies across levels of the covariate. Thus, the interaction model was used to summarize the observed hormone patterns in each category, and the main effects model formally tested for mean hormone concentration differences between categories using an F-Test, that is, assuming no interactions, the significance of a covariate taking into account the effects of days since LMP can by formally tested using an F-test. The area under the common regression spline curve was calculated by summing the estimated spline values at days 1–28; variances were computed using the standard delta method. Statistical significance of the difference in the overall area under the curves, and for the follicular and luteal phases, was estimated from analyses including total days since LMP and for days 1–14 and 15–28, respectively. Additional adjustment assessed whether there were independent associations of anthropometric, reproductive, and lifestyle factors with the same hormone. These models simultaneously included factors that were associated with hormones in univariate models.

## 3. Results

Table 1 shows the characteristics of the study subjects and mean hormone concentrations. On average, women were 35 years of age with a BMI of 25.3 kg/m^2^. About two-thirds of women were born in Ulaanbaatar, had a college education, lived in an apartment or house, and exercised vigorously. All women were parous and, on average, breastfed for 19.3 months per child. Average age at menarche was 14.8 years. Estrone and estradiol concentrations were highly correlated (r = 0.93) and androstenedione and testosterone were moderately correlated (r = 0.79); all other correlations between hormones were r < 0.50).

**Figure 1:**
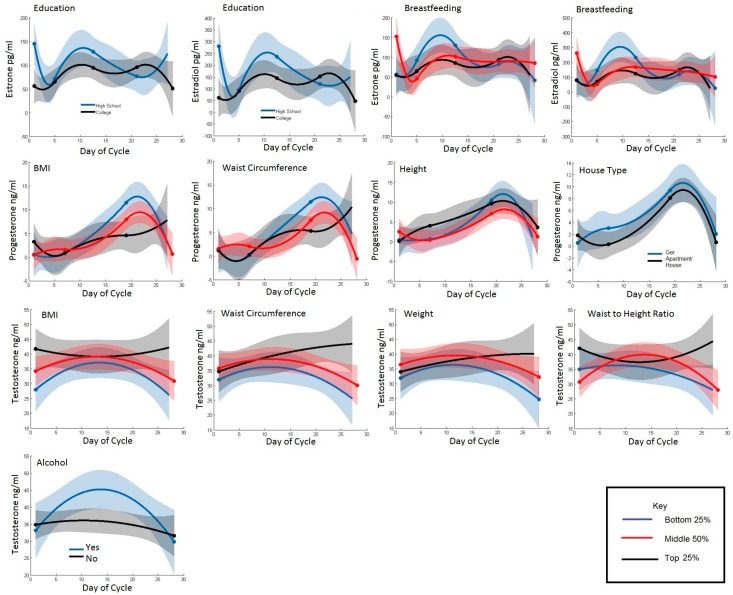
T Hormones concentrations are shown over the menstrual cycle in Mongol women according to different lifestyle (education, ger and alcohol), reproductive (breastfeeding) and anthropometric (BMI, waist circumference, height, weight, waist-to-height ratio). Distributions of the risk factors are cut into the top 25% (black), the middle 50% (red), and the bottom 25% (blue). Estrone and an estradiol are shown in row 1, progesterone is shown is row 2, and testosterone is shown in rows 3 and 4.

Table 2 displays the mean concentration of each hormone over the menstrual cycle by categories (bottom 25%, middle 50%, and top 25% for continuous variables) of lifestyle, reproductive and anthropometric factors. Figure 1 displays plots of the same categories of the hormone concentrations by day since LMP for select lifestyle, anthropometric, and reproductive characteristics to allow visual inspection of the associations that reached statistical significance either over the whole menstrual cycle or during a specific phase.

Characteristics indicative of a more urban (i.e., higher education, greater adiposity, permanent housing) rather than traditional lifestyle were associated with lower estrogen and progesterone concentrations and higher testosterone concentrations. Mean estrone and estradiol concentrations over the entire menstrual cycle in college-educated women were 19.8% and 26.3% lower, respectively, than in women who were high-school educated (*p* = 0.02). This difference appeared to be driven by a more pronounced peak during days 1–14 of the menstrual cycle among high-school educated women. Progesterone concentrations across the menstrual cycle were 55.8% lower in women who lived in an apartment or house compared with women who lived in a traditional dwelling (ger), but this association did not reach statistical significance (Table 2, *p* = 0.08). Testosterone concentrations were higher among women with greater and more central adiposity. Mean testosterone over the entire menstrual cycle was 13.9%, 23.7%, 22.1%, and 18.3% greater in the highest compared to lowest quarter of weight, BMI, waist circumference, and waist-to-height ratio categories, respectively. The general pattern of testosterone over the cycle was bell-shaped with increasing concentrations in the follicular phase and decreasing concentrations in the luteal phase. However, among women in the highest categories of weight and waist circumference, testosterone continued to increase in the luteal phase instead of declining; among women with the highest BMI, testosterone concentrations remained consistently higher across the entire cycle. Androgen concentrations, but not estrogens or progesterone, were positively associated with alcohol consumption; women who drank alcohol (mostly in the form of airag, fermented mare’s milk) had 14.4% and 13.8% higher testosterone and androstenedione, respectively, compared with those that did not. Alcohol intake did not differ by housing type or education (data not shown).

Considering the variation in hormone concentrations by menstrual cycle phase, we also tested if there were differences in hormones according to lifestyle, anthropometric, or reproductive factors specific to either the follicular or luteal phase of the cycle. Estrone and estradiol between days 1–14 were inversely associated with duration of breastfeeding per child. Women who breastfed less than 9.5 months per child had higher follicular estrone (97.2%; *p* = 0.04) and estradiol (239.1%; *p* = 0.006) than women who breastfed for the longest durations. Progesterone was positively associated with height between days 1–14, when progesterone concentrations are at their lowest. The tallest women had 176.3% higher progesterone compared with the shortest. Progesterone was inversely associated with adiposity between days 15–28. Among women with the highest BMI and waist circumference, midluteal peak progesterone was 56.5% and 50.4% lower than in women in the lowest category.

We attempted to determine which of the observed associations with hormones were independent (Table 3). Mutual adjustment attenuated some of the associations. Breastfeeding duration per child was longer among college-educated women than high school-educated women (20.5 vs. 17 months; *p* = 0.03). With simultaneous adjustment for education and breastfeeding, the association of education with whole-cycle estrogens remained similar; however; the association of breastfeeding with follicular estrogens was substantially attenuated (difference between long vs. short breastfeeding duration for estradiol decreased from 239.1% to 63.7%). Ger-dwelling women were on average 2 cm shorter than women who lived in a house/apartment (*p* = 0.003), thus the positive association between height and follicular phase progesterone was stronger after adjusting for ger living (difference between height > 161 cm vs. <154 cm increased from 176.3% to 236.5%). The percent differences in progesterone associated with less central adiposity and living in a ger were only slightly attenuated with mutual adjustment (difference in waist circumference > 88 cm vs. <72 cm decreased from 50.4% to 43.3%; difference between living in a house or apt vs. ger decreased from 55.8% to 42.2%). The association between alcohol intake and higher testosterone was attenuated after adjusting for adiposity markers (difference in testosterone between drinkers vs. nondrinkers decreased from 14.4% to 10.7%), and the percent differences in testosterone associated with adiposity (except for weight) were only slightly attenuated with adjustment for alcohol.

## 4. Discussion

The present study demonstrates that Mongol women with a more urban lifestyle had lower premenopausal estrogen and progesterone, and higher testosterone concentrations than Mongol women with a more traditional lifestyle. In particular, heavier, more-educated women who were residing in modern buildings had higher circulating testosterone, and lower progesterone and estrogen concentrations, respectively, than their leaner, less-educated, ger-dwelling counterparts. Greater height, shorter duration of breastfeeding, and alcohol use were associated with higher progesterone, estrogens, and testosterone, respectively. Other established or putative breast cancer risk factors, such as younger age at menarche, lower parity, and lower levels of physical activity were not associated with premenopausal hormone levels.

Socioeconomic circumstances appeared to account for a proportion of the variation we observed in estrogen concentrations within the Mongol women. Few, if any, studies have directly quantified the association between markers of socioeconomic status and pre- or post-menopausal estrogen concentrations. A longitudinal and multiethnic USA study reported no association between educational status and estrogen concentrations during the early menopausal transition [21]. Within Mongolia, it appears that as women’s socioeconomic status rises through higher educational attainment, their estrogen concentrations decline, which is not explained by the increases in breastfeeding associated with higher education. In contrast, education explained a substantial portion of the difference in follicular-phase estrogen concentrations according to breastfeeding duration. The modest but persistent inverse association between breastfeeding and estradiol in the follicular phase is provocative given that there is only one study to observe an association between estrogen concentrations and increased premenopausal breast cancer measured estrogens in the follicular phase [22]. Unlike ours, other studies have found no association between breastfeeding duration and estrogens among premenopausal Western and Japanese women [23,24], perhaps due to the timing of the measurements and/or narrower distributions of breastfeeding duration. Deciphering the mechanism beneath the socioeconomic/estrogen relationship in economically developing populations may help identify novel, nonreproductive breast cancer risk factors.

Unique aspects of the Mongolian lifestyle were associated, albeit weakly, with patterns of premenopausal progesterone concentrations. In particular, Mongol women living in a ger appeared to have higher progesterone levels than women living in housing that is more modern, but this finding was not statistically significant. We previously reported higher progesterone concentrations in women born outside of Ulaanbaatar than women born in Ulaanbaatar [15]. Women living in gers, which tend to be erected in the city’s outskirts by recent immigrants to Ulaanbaatar, were also more likely to have previously lived as a nomad, and to have been born in the countryside [14]. Thus, ger living is likely a proxy for other, unmeasured factors related to a traditional Mongolian lifestyle and lower breast cancer risk. While the role of progesterone in breast cancer etiology is less studied than that of estrogens, a pooled analysis of seven prospective studies reported higher concentrations in premenopausal controls than cases between days 15–18 [6]. This is consistent with the higher midcycle progesterone concentrations that we observed in women with a more traditional lifestyle, and their apparently lower breast cancer incidence rates. 

Adiposity was positively associated with androgen concentrations in our data, as in studies of premenopausal women from both Western [25,26] and Asian populations [23]. Additionally, women with upper body or central obesity have higher androgen production and testosterone concentrations, whereas women with lower body obesity have higher estrone concentrations from peripheral aromatization of androgens [27]. In line with this, waist-to-height ratio, a measure of central adiposity, was positively associated with androgens in our study. Our findings further suggest that the pattern of testosterone production may differ over the menstrual cycle with regard to adiposity and fat distribution, with those women having the highest levels of weight and waist circumference showing a persistently elevated testosterone concentration during the second half of the menstrual cycle.

Higher breast cancer rates among Western than Asian women has been speculated to be explained by differences in reproductive and dietary behaviors which, in turn, are hypothesized to result in higher estrogen concentrations in populations at elevated risk. Much of the support for these hypotheses comes from studies of postmenopausal women, while evidence is less consistent in premenopausal women. However, if hormone exposure over time or during critical windows is important, than premenopausal hormone profiles may be relevant for both pre- and post-menopausal breast cancer risk. We previously demonstrated that Mongol women, who have very low breast cancer rates, have, on average, substantially higher premenopausal estradiol and progesterone concentrations, and lower testosterone than higher risk women in the UK [15]. Our current investigation into the associations between hormones and breast cancer risk factors in the Mongolian population suggests that women with more urban lifestyles—marked by higher education, living in modern housing, and greater adiposity—have a different hormone profile than women with a traditional Mongolian lifestyle, and that the direction of the difference is toward what we’ve previously observed in British women. These findings continue to support a role for hormones in breast cancer carcinogenesis, but suggest the importance of testosterone and progesterone.

Limitations of our study include collecting samples at a single time point without restriction to the day of cycle, although this allowed us to assess hormones over the entire menstrual cycle, as well as during each phase. This method does not allow accounting for the intraindividual variation in hormones during a single cycle, or intraindividual variation over multiple cycles, and if this variation were larger than the between-woman variation in hormones, it would limit our ability to detect differences. To the extent possible, we attempted to study a representational sample of premenopausal, Mongol women, although factors that both resulted in study participation and were related to hormone concentrations may have affected our findings. For example, all women were parous, limiting the generalizability of the findings to nulliparous women. Further exploration of the association of alcohol intake and androgen concentrations was limited because airag, homemade fermented mare’s milk, varies considerably in the amount of alcohol it contains. While we did not adjust for the multiple comparisons conducted in this exploratory investigation, replication in other populations undergoing rapid lifestyle changes are possible given the feasibility of collecting untimed samples. Finally, confirmation of our interpretation of our findings will require directly evaluating the associations of the hormones with breast cancer incidence within this population.

## 5. Conclusions

Our results suggest that within Mongolia, women with an urban lifestyle have higher androgen concentrations and lower estrogens and progesterone than women with a more traditional lifestyle. Over the last decade and a half, Mongolia has experienced profound economic changes resulting in mass migration from a nomadic or seminomadic existence to a more urban lifestyle in the capital city of Ulaanbaatar. Together with the contrast in risk factors and breast cancer incidence rates between traditional and urban settings, our findings suggest that prevailing hypotheses that link hormonal factors with established risk factors may be more complicated than previously thought. Studies that consider both biological and sociocultural factors in Asian countries at varying risk for breast cancer could be fruitful for testing whether current risk factors hold across populations and for generating new, more complex hypotheses.

## Figures and Tables

**Table 1:** Demographic, lifestyle, reproductive and anthropometric characteristics, and hormone concentrations among 314 premenopausal Mongol women.

Characteristic	N	Mean (SD) or Percent	Min	Max
**Lifestyle and Demographic**				
Age (y)	314	34.9 (4.8)	24.0	45.0
Education				
Some/graduated high school	110	35%		
Some/graduated college	204	65%		
House Type				
Ger (yurt)	103	33%		
Apartment or House	211	67%		
Previously lived as nomad	41	13%		
Physical Activity Level				
Low	52	16.6%		
Moderate	77	24.5%		
Vigorous	184	58.6%		
Ever Smokers	54	17.2%		
Consume Alcohol	70	22.3%		
**Reproductive**				
Age at menarche (y)	314	14.8 (1.5)	11.0	20.0
Total number of months breastfed	314	39.2 (30.7)	0.0	168
Average months breastfed each child	314	19.3 (13.9)	0.0	72.0
**Anthropometrics**				
Weight (kg)	314	62.6 (12.0)	35.8	119
Height (cm)	314	157.2 (5.6)	138.6	172
Body mass index (kg/m^2^)	314	25.3 (4.5)	16.4	46.3
Waist circumference (cm)	314	81.7 (11.0)	57.0	121
Weight at 18 (kg)	313	54.4 (7.6)	37.0	78.0
BMI at 18 (kg/m^2^)	313	22.0 (3.1)	15.2	34.8
**Hormones**				
Androstenedione (ng/mL)	314	1.0 (0.4)	0.3	2.50
Testosterone (ng/dL)	314	32.1 (13.6)	9.9	103
Progesterone (ng/mL)	297	5.1 (6.9)	0.1	33.3
Estrone (pg/mL)	314	85.1 (50.1)	18.5	458
Estradiol (pg/mL)	314	133 (103)	7.5	995

**Table 2:** Mean (SD) age-adjusted serum hormone concentrations over the menstrual cycle by anthropometric, reproductive, and lifestyle characteristics.

Characteristic	n	Androstenedione ng/mL	Testosterone ng/dL	Progesterone ng/mL	Estrone pg/mL	Estradiol pg/mL
Lifestyle and Demographic						
Age (Years)						
24–31	61	1.2 (0.05)	35.0 (1.8)	1.1 (0.9)	90.4 (10.5)	147 (24)
31–39	174	0.9 (0.05)	29.8 (1.9)	2.3 (1.1)	94.5 (14.1)	165 (32)
39–45	79	0.7 (0.06)	25.3 (2.0)	2.4 (1.0)	90.0 (11.6)	151 (26)
*p*		<0.01	<0.01	0.36	0.89	0.69
Education						
High School	110	1.2 (0.06)	35.7 (2.2)	2.1 (1.2)	105 (16)	178 (35)
College	204	1.1 (0.05)	33.6 (1.6)	1.0 (0.8)	84.3 (9.1)	131 (21)
*p*		0.22	0.20	0.13	0.02	0.02
House Type						
Ger	103	1.1 (0.07)	35.0 (2.3)	2.5 (1.3)	97.1 (16.2)	164 (37)
Apartment/House	211	1.1 (0.05)	34.1 (1.7)	1.1 (0.8)	91.5 (9.5)	146 (22)
*p*		0.53	0.62	0.08	0.56	0.42
Previously lived as Nomad						
No	41	1.1 (0.06)	34.4 (2.0)	1.5 (1.2)	95.1 (14.8)	156 (34)
Yes	273	1.1 (0.07)	34.6 (2.3)	0.4 (1.1)	78.6 (12.8)	118 (29)
*p*		0.44	0.92	0.29	0.20	0.19
Physical Activity Level						
Low	52	1.1 (0.08)	34.4 (2.6)	0.5 (1.4)	84.2 (17.3)	134 (39)
Moderate	77	1.1 (0.07)	34.7 (2.4)	2.0 (1.1)	98.9 (13.9)	160 (31)
Vigorous	184	1.1 (0.06)	34.3 (2.1)	1.6 (1.0)	94.7 (12.0)	156 (27)
*p*		0.99	0.98	0.38	0.56	0.66
Smoking						
No	260	1.1 (0.06)	34.2 (2.1)	1.4 (1.0)	93.5 (11.8)	151 (27)
Yes	54	1.1 (0.08)	35.2 (2.6)	1.8 (1.4)	91.6 (17.9)	157 (41)
*p*		0.97	0.64	0.71	0.87	0.81
Alcohol						
No	244	1.2 (0.07)	38.3 (2.5)	1.3 (1.3)	104 (17)	160 (39)
Yes	70	1.1 (0.05)	33.4 (1.8)	1.5 (0.9)	90.9 (10.5)	150 (24)
*p*		0.01	0.01	0.87	0.22	0.68
Anthropometrics						
Weight (kg)						
36–55	81	1.1 (0.05)	31.2 (1.9)	2.0 (0.9)	87.8 (10.6)	153 (24)
55–70	159	1.2 (0.06)	35.6 (2.1)	1.8 (1.2)	98.3 (15.5)	163 (35)
70+	74	1.1 (0.06)	35.5 (1.9)	0.4 (0.9)	90.4 (11.0)	132 (25)
*p*		0.18	0.05	0.23	0.56	0.45
Height (cm)						
139–153	75	1.2 (0.06)	35.3 (1.9)	1.6 (0.9)	91.4 (10.8)	148 (25)
153–161	161	1.1 (0.06)	32.6 (2.1)	0.6 (1.2)	91.7 (15.5)	149 (35)
161+	78	1.2 (0.06)	36.3 (1.9)	2.4 (0.9)	95.9 (10.9)	157 (25)
*p*		0.06	0.11	0.12	0.92	0.94
Body mass index (kg/m^2^)						
16–22	48	1.1 (0.05)	31.1 (1.8)	2.4 (0.9)	91.0 (10.6)	153 (24)
22–28	218	1.1 (0.06)	34.7 (2.1)	1.3 (1.2)	85.7 (15.5)	138 (35)
28+	48	1.2 (0.06)	38.4 (1.9)	0.8 (0.9)	107 (11)	171 (25)
*p*		0.26	<0.01	0.25	0.16	0.40
Waist circumference (cm)						
57–73	74	1.1 (0.05)	31.2 (1.8)	2.5 (0.9)	89.7 (10.6)	160 (24)
73–89	161	1.1 (0.06)	34.6 (2.1)	1.3 (1.2)	95.5 (15.5)	159 (35)
89+	79	1.2 (0.06)	38.1 (2.0)	0.7 (0.9)	92.6 (11.4)	131 (26)
*p*		0.15	0.01	0.19	0.86	0.53
Waist/Height Ratio (cm/cm)						
0.35–0.47	85	1.1 (0.05)	32.5 (1.9)	1.8 (0.9)	88.7 (10.6)	151 (24)
0.47–0.56	148	1.1 (0.06)	34.1 (2.1)	1.5 (1.2)	88.6 (15.5)	144 (35)
0.56+	81	1.2 (0.06)	38.5 (1.9)	1.0 (0.9)	107 (11)	166 (25)
*p*		0.51	0.02	0.71	0.22	0.67
Weight at 18 (kg)						
37–48	56	1.1 (0.05)	33.8 (1.8)	0.9 (0.9)	89.4 (10.4)	139 (24)
48–60	164	1.1 (0.06)	34.8 (2.1)	1.8 (1.2)	95.7 (15.1)	159 (34)
60+	93	1.0 (0.06)	34.0 (2.1)	0.8 (1.0)	88.4 (12.0)	142 (27)
*p*		0.33	0.85	0.46	0.75	0.64
BMI at 18 (kg/m^2^)						
<20	48	1.2 (0.06)	34.9 (1.9)	1.5 (0.9)	94.9 (10.8)	151 (24)
20–24	218	1.1 (0.06)	34.0 (2.1)	1.5 (1.2)	89.4 (15.2)	144 (35)
24+	48	1.1 (0.06)	35.9 (2.0)	1.3 (0.9)	104 (11)	179 (25)
*p*		0.41	0.62	0.98	0.43	0.38
Reproductive						
Age at menarche (years)						
11–14	58	1.1 (0.05)	33.4 (1.6)	1.1 (0.8)	88.9 (15.4)	144 (21)
14–16	155	1.2 (0.06)	35.5 (2.2)	1.8 (1.2)	97.1 (15.4)	159 (35)
16–18	101	1.1 (0.08)	35.0 (2.8)	1.3 (1.3)	93.7 (15.8)	140 (36)
*p*		0.26	0.43	0.61	0.68	0.72
Parity (number of live births)						
1	36	1.2 (0.06)	35.0 (2.1)	1.3 (1.2)	92.4 (15.5)	148 (35)
1+	278	1.1 (0.06)	33.6 (1.9)	1.6 (0.9)	94.4 (10.9)	157 (25)
*p*		0.12	0.48	0.78	0.85	0.72
Average months breastfed per child (months)						
<9.5	78	1.1 (0.06)	35.2 (1.9)	2.0 (0.9)	99.3 (10.8)	167 (24)
9.5–24	136	1.1 (0.06)	34.5 (2.2)	1.3 (1.2)	98.9 (15.8)	165 (36)
24–72	100	1.1 (0.06)	33.8 (1.9)	1.2 (0.9)	84.8 (10.8)	131 (25)
*p*		0.94	0.82	0.67	0.37	0.32

**Table 3:** Percent differences in hormones by characteristics of women before and after mutual adjustment.

Hormone Cycle Phase	Characteristic	Unadjusted Model		Mutually-Adjusted Model	
		% Difference ^a^	*p*	% Difference ^a^	*p*
**Estrone**					
Whole Cycle	Education (College vs. High school)	19.8	0.02	18.2	0.03
Follicular	Breastfeeding (<9.5 vs. 24+ months)	97.2	0.02	40.6	0.07
**Estradiol**					
Whole Cycle	Education (College vs. High school)	26.3	0.02	23.6	0.03
Follicular	Breastfeeding (<9.5 vs. 24+ months)	239.1	0.003	63.7	0.01
**Progesterone**					
Whole Cycle	House type (House or Apartment vs. Ger)	55.8	0.08	42.3 ^b^	0.10
Follicular	Height (161+ vs. <153 cm)	176	0.01	237 ^c^	0.02
Luteal	Waist circumference (89+ vs. <73 cm)	50.4	0.02	43.4 ^c^	0.02
**Testosterone**					
Whole Cycle	Weight (70+ vs. <55 kg)	13.9	0.05	11.0 ^d^	0.07
Whole Cycle	BMI (28+ vs. <22 kg/m^2^)	23.7	0.001	17.1 ^d^	0.001
Whole Cycle	Waist circumference (89+ cm vs. <73 cm)	22.1	0.002	17.9 ^d^	0.005
Whole Cycle	Waist to height ratio (0.56+ vs. <0.47)	18.3	0.007	15.3 ^d^	0.01
Whole Cycle	Alcohol (Yes vs. No)	14.4	0.009	10.7 ^e^	0.01

^a^ % difference is relative; ^b^ adjusted for height and waist; ^c^ adjusted for house type only; ^d^ adjusted for alcohol only; ^e^ adjusted for BMI, waist circumference, waist to height ratio.
